# Vericiguat in heart failure with reduced ejection fraction: not vanity, but vindication

**DOI:** 10.1093/eschf/xvaf044

**Published:** 2026-01-18

**Authors:** Andrew P Ambrosy

**Affiliations:** Department of Cardiology, Kaiser Permanente San Francisco Medical Center, San Francisco, CA, USA; Division of Research, Kaiser Permanente Northern California, Pleasanton, CA, USA

**Keywords:** HFrEF, Vericiguat, Outcomes

Vericiguat, a soluble guanylate cyclase stimulator, augments cyclic guanosine monophosphate signalling to promote vasodilation and blunt adverse cardiac remodelling in patients with heart failure with reduced ejection fraction (HFrEF).^1^ In the phase 3 VICTORIA trial, the addition of vericiguat to background guideline-directed medical therapy (GDMT) significantly reduced the composite endpoint of cardiovascular (CV) death or first HF hospitalization in patients with a recent worsening HF event, leading to Food and Drug Administration approval in 2021 for adults with symptomatic chronic HFrEF and an ejection fraction below 45%.^2,3^ Yet the therapy received only a Class IIb, Level of Evidence B-R guideline recommendation.^4^ This cautious endorsement reflected several concerns: the benefit was largely driven by a reduction in HF hospitalizations rather than CV mortality, patients with the highest natriuretic peptide levels appeared to derive little or no benefit, and the study was largely conducted in a pre-sodium-glucose cotransporter-2 (SGLT2) inhibitor era. Against this backdrop, the VICTOR trial was launched to evaluate the efficacy and safety of vericiguat in a broader population of patients with HFrEF (≤40%) without recent worsening HF event receiving maximally tolerated GDMT.^5^

The VICTOR trial enrolled 6105 patients randomized to vericiguat or placebo. Participants had a mean age of 67 years, just under one-quarter were women, and nearly two-thirds were White.^6^ The mean left ventricular ejection fraction was 30%, with most patients reporting New York Heart Association class II symptoms. Strikingly, about half of the cohort had never been hospitalized for HF, and nearly one in three were not receiving diuretics. This was an exceptionally well-treated population by contemporary standards: 78% received a mineralocorticoid receptor antagonist (MRA), 56% an angiotensin receptor–neprilysin inhibitor (ARNi), and 59% a SGLT2 inhibitor, with 44% achieving quadruple therapy. In this context, vericiguat did not reduce the primary composite endpoint of CV death or HF hospitalization among patients with HFrEF and no recent worsening.^7^ However, nominal reductions in CV mortality were observed in the vericiguat group compared with placebo.

The initial reaction from the European Society of Cardiology community and the lay press was that VICTOR was a neutral trial—one that missed its primary endpoint with only a chance signal for reduced CV death. Yet a closer look at the data tells a very different story. Although natriuretic peptide levels rose over time in both treatment arms, the trajectory was significantly attenuated with vericiguat compared with placebo, suggesting that if the drug does not induce reverse remodelling, it at least slows adverse remodelling.^8^ In this stable ambulatory HFrEF cohort, 59% of first worsening HF events were outpatient worsening HF episodes defined by oral diuretic initiation or intensification. These events carried a heightened risk of subsequent mortality, and when the composite endpoint was broadened to include outpatient worsening HF, vericiguat produced a statistically significant reduction in events, irrespective of whether all-cause death was included.^8^ Real-world evidence reinforces this observation, as outpatient worsening HF has been shown to represent the majority of worsening HF events and to portend poor short-term outcomes.^9^

The mortality findings, although technically hypothesis-generating within the prespecified statistical hierarchy, are far from spurious. VICTOR was designed to accrue nearly 600 CV deaths, providing 80% power to detect a hazard ratio of 0.80 for this endpoint.^10^ In fact, reductions were observed in both sudden cardiac and HF-related deaths, a pattern that is biologically plausible given the disease state and vericiguat’s mechanism of action. All-cause mortality was also reduced—a rare and coveted achievement in CV drug development, seen consistently across the four foundational therapies for HFrEF. These results are further bolstered by an individual participant pooled analysis of VICTOR and VICTORIA, which demonstrated consistent benefits of vericiguat on the composite of CV death or HF hospitalization, as well as on the individual components, across all major subgroups.^11^ Taken together, this is a familiar arc in HF therapeutics: early beneficial haemodynamic effects, reflected in a more favourable natriuretic peptide trajectory, followed by fewer worsening HF events, and ultimately translating into improvements in CV and all-cause mortality.

The central question now is how best to implement vericiguat in contemporary practice, given the expanding therapeutic armamentarium that includes four foundational classes of drugs and several second-line agents.^12^ Vericiguat has been studied in more than 11 000 patients across the spectrum of acuity, from stable to recently decompensated, and has consistently proven safe, well tolerated, and efficacious for both fatal and nonfatal outcomes. This robust evidence base warrants a shift from the current Class IIb, Level of Evidence B-R guideline designation to at least a Class IIa, Level of Evidence A recommendation. Indeed, one could make a cogent argument for a Class I recommendation, as vericiguat would otherwise be the only therapy with a demonstrated mortality benefit to lack one.

Equally important, vericiguat should not be relegated to the status of a ‘fifth pillar.’ First, it reduced CV death in a stable HFrEF population receiving excellent background medical therapy, dispelling the myth of the ‘stable’ HF patient when the first event for many is sudden cardiac death. Second, while guidelines promote quadruple therapy, this standard is rarely achieved in practice due to patient-, provider-, and system-level barriers.^13,14^ Third, sequencing of therapies is already individualized, and vericiguat is uniquely positioned given its haemodynamic neutrality and absence of adverse effects on renal function or potassium, which often limit initiation and uptitration of other agents. For these reasons, restricting vericiguat to patients who ‘fail’ GDMT or who are ‘progressing’ despite optimal therapy would be a mistake.

At the same time, special populations may stand to benefit most, including those with borderline blood pressure,^15^ prior intolerance of GDMT,^16^ or advanced non-dialysis-dependent kidney disease.^17^ Evidence gaps remain—particularly around patient-reported outcomes. In VICTORIA, health-related quality of life improved to a similar extent in both arms after a recent worsening event, with no significant difference between groups.^18^ By contrast, in VICTOR, stable HFrEF patients treated with vericiguat appeared to experience less deterioration compared with placebo. The impact of vericiguat on functional outcomes also remains uncertain, as it did not improve 6-min walk distance in patients with HF with preserved EF (HFpEF), a population in which comorbidities frequently drive functional limitations.^19^ Finally, cost-effectiveness analyses should be undertaken to inform payers and policymakers. Still, while cost and cost-effectiveness are relevant considerations, they should not overshadow the compelling evidence of vericiguat’s safety, tolerability, and efficacy.

Taken together, the evidence from VICTORIA, VICTOR, and pooled analyses provides compelling proof that vericiguat delivers meaningful clinical benefit across the spectrum of HFrEF. These findings justify upgrading its guideline status from Class IIb, Level of Evidence B-R to at least Class IIa, Level of Evidence A. The benefits of vericiguat extend across the risk continuum, underscoring that it should not be reserved only for patients who ‘fail’ or ‘progress’ on quadruple therapy. While further research is warranted to define its impact on quality of life, functional capacity, and cost-effectiveness, these gaps should not obscure the robust and reproducible efficacy, safety, and tolerability already demonstrated. As we move towards an era of personalized medicine in HFrEF, the familiar four pillars may soon give way to five building blocks, with vericiguat firmly established as part of the foundation. In the ongoing debate over vericiguat’s place in therapy, the weight of evidence is clear: this is not vanity, but vindication.

## Data Availability

No data were generated or analysed for this manuscript.
